# Resolving Endoplasmic Reticulum‐Protein Misfolding Restores Corticosteroid Sensitivity in Experimental Models of Severe Asthma

**DOI:** 10.1111/all.70353

**Published:** 2026-04-26

**Authors:** Prabuddha S. Pathinayake, Alexandra C. Brown, Nikhil T. Awatade, Philip M. Hansbro, Katherine J. Baines, Richard Y. Kim, Nathan W. Bartlett, Jay C. Horvat, Peter A. B. Wark

**Affiliations:** ^1^ Infection Research Program, Hunter Medical Research Institute and School of Biomedical Sciences and Pharmacy University of Newcastle Newcastle New South Wales Australia; ^2^ Immune Health Program Hunter Medical Research Institute and School of Biomedical Sciences and Pharmacy, University of Newcastle Newcastle New South Wales Australia; ^3^ Immune Health Program Hunter Medical Research Institute and School of Medicine and Public Health, University of Newcastle Newcastle New South Wales Australia; ^4^ Centre for Inflammation, Centenary Institute, and University of Technology Sydney, Faculty of Science, School of Life Sciences Sydney New South Wales Australia; ^5^ University of Technology Sydney, Faculty of Science, School of Life Sciences Sydney New South Wales Australia; ^6^ Department of Respiratory and Sleep Medicine John Hunter Hospital Newcastle New South Wales Australia; ^7^ School of Translational Medicine Monash University Melbourne Victoria Australia; ^8^ Respiratory Medicine Alfred Hospital Melbourne Victoria Australia

**Keywords:** 4‐PBA, ER stress, inflammation, protein misfolding, severe asthma

## Abstract

**Background:**

People with severe steroid‐resistant asthma are refractory to treatment with the mainstay inhaled corticosteroids (ICS), emphasising the urgent need for alternative therapies. This study aimed to assess the effect of endoplasmic reticulum stress (ERS) on steroid responsiveness and to evaluate the efficacy of 4‐phenylebuteric acid (4‐PBA) as an add‐on treatment to restore steroid responses in severe asthma.

**Methods:**

The relationship between ERS and steroid response was assessed by treating human airway epithelial cells (AEC) with chemical ERS inducers or TNF, IFN‐γ, and IL‐17, and assessing the effects of dexamethasone (Dex) and 4‐PBA. The correlation between genes associated with ERS and GR‐signalling was assessed in sputum cells from patients with severe asthma, and the effects of 4‐PBA were assessed in two murine models of severe, steroid‐resistant asthma.

**Results:**

Chemical ERS inducers significantly downregulated the expression of corticosteroid‐responsive genes, HSD11B2 and FKBP5 and reduced GR nuclear translocation in basal AECs. Treatment with TNF, IFN‐γ and IL‐17 upregulated ERS and protein misfolded markers while reducing Dex‐induced GR nuclear translocation. In sputum cells from patients with severe asthma, ERS genes negatively correlated with GR‐signalling. In differentiated primary bronchial epithelial cells (pBECs), treatment with 4‐PBA reversed TNF, IFN‐γ and IL‐17‐induced steroid resistance by upregulating HSD11B2 and FKBP5 gene expression and downregulating inflammatory genes. 4‐PBA together with Dex significantly reduced airway inflammation and/or AHR in experimental models of severe, steroid‐resistant asthma.

**Conclusion:**

We provide evidence for ERS inducing steroid resistance that underpins severe asthma and demonstrate a therapeutic potential for restoring steroid sensitivity in severe asthma with 4‐PBA.

## Graphical abstract

This study investigates the role of ER stress in steroid resistance in severe asthma. Results from advanced 3D models of human bronchial epithelial cells, steroid‐resistant severe asthma mouse models, and human severe asthma sputum samples show that treatment with ICS in combination with the ER stress inhibitor 4‐PBA significantly enhances steroid responses. This is demonstrated by increased expression of steroid‐responsive genes, decreased expression of inflammatory genes, enhanced GR nuclear translocation, reduced inflammatory cell counts in the lungs of murine models, and improved lung function. 4‐PBA, 4‐phenylbutyric acid; ATF6, activating transcription factor 6; BALF, bronchoalveolar lavage fluid; CMU, Chlamydia muridarum; CXCL10, C‐X‐C motif chemokine ligand 10; ER, endoplasmic reticulum; FKBP5, FK506‐binding protein 5; GR, glucocorticoid receptor; Hinf, non‐typeable Haemophilus influenzae; HSD11B2, hydroxysteroid 11‐beta dehydrogenase 2; ICS, inhaled corticosteroids; IL, interleukin; OVA, ovalbumin; pBECs, primary bronchial epithelial cells; SRAAD, severe corticosteroid‐resistant allergic airways disease.

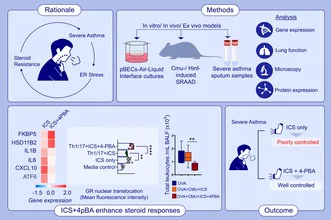

## 1 Introduction

Asthma is a heterogeneous chronic inflammatory disease of the airways with various endotypes and phenotypes [1]. Inhaled corticosteroids are the mainstay treatment for asthma. They control symptoms and reduce the risk of acute exacerbations in the majority of people with asthma at low to medium doses [2]. However, between 5% and 25% of patients with asthma suffer from the disease that is not adequately controlled, despite optimal doses of inhaled corticosteroids and long‐acting bronchodilators, experiencing poor symptom control and frequent exacerbations [3, 4]. Severe asthma, while heterogeneous in the majority of people, displays features of chronic airway inflammation refractory to inhaled corticosteroids. Those with the most severe disease, similar to those with mild asthma, have inflammation characterised by type‐2 high inflammation associated with increased IL‐4, IL‐13 and IL‐5. This leads to airway eosinophilia and is associated with allergy, but unlike those with mild disease, this inflammation persists despite inhaled corticosteroid treatment [5, 6, 7]. These individuals have inherent corticosteroid‐refractory responses with persistent T‐helper type‐2 (TH2) responses [8, 9]. A significant proportion also demonstrates inflammation that is not TH2, characterised by elevated airway neutrophils and activation of TH1 and TH17 responses [10], which is also inherently refractory to inhaled corticosteroids. In those with severe TH2 asthma, biologic therapies targeting these pathways are effective at overcoming this persistent inflammation and improving disease control but require lifelong treatment and are expensive [11, 12]. Unlike TH2 asthma, there are currently no biologics approved specifically for severe TH1/TH17 asthma. Currently, only Tezepelumab (anti‐TSLP) is available to use in severe asthma without TH2 high disease [13, 14]. Understanding the molecular mechanisms behind corticosteroid‐refractory inflammation in severe asthma is crucial in developing novel therapies, including returning them to a state that will respond to low to medium‐dose inhaled corticosteroids.

Glucocorticoids are used to treat chronic inflammatory diseases. They bind to Glucocorticoid Receptors (GR), which belong to the nuclear receptor superfamily of transcription factors [15]. GR predominantly resides in the cytoplasm of cells as part of a large multi‐protein complex that includes chaperone proteins (hsp90, hsp70 and p23) and immunophilins of the FK506 family (FKBP51 and FKBP52) [16]. These accessory proteins are crucial in maintaining the receptor in a transcriptionally inactive conformation, while favouring high‐affinity ligand binding and GR nuclear translocation. When glucocorticoids bind with the GR, accessory proteins dissociate from the complex, and the activated GR rapidly translocates into the nucleus through nuclear pores [17]. Once inside the nucleus, GR binds with glucocorticoid‐responsive elements (GREs) and regulates the expression of various target genes including those that encode anti‐inflammatory factors [18]. Due to their broad‐spectrum anti‐inflammatory properties, synthetic glucocorticosteroids have been widely used to treat various chronic inflammatory diseases, including asthma.

Current knowledge on cellular and molecular mechanisms that underpin corticosteroid resistance in severe asthma is limited [4, 19]. Some investigators have described reduced GR‐α expression [20], impaired GR nuclear translocation [21], and defective GR‐corticosteroid or GR‐DNA binding [22]. GR‐α has been shown to be repressed by increased GR‐β, resulting in reduced HDAC2 activity and p38 MAP kinase‐induced GR phosphorylation [23], along with exaggerated NLRP3 activity [24], and this has been described to result in corticosteroid insensitivity in asthma. We previously showed that endoplasmic reticulum (ER) stress (ERS) and the unfolded protein response (UPR) are elevated in the airways of people with severe asthma [25]. The ER is responsible for folding secretory and transmembrane proteins and ensures protein folding quality [26]. Excessive ERS triggered by disturbed cellular mechanisms could lead to the accumulation of aberrantly folded proteins in the ER, resulting in the initiation of an adaptive compensatory response, termed the UPR. The UPR involves recruitment of protein‐folding chaperones/co‐chaperones to enhance protein folding, degrade terminally misfolded proteins and reduce protein translation and if unresolved, results in cell death [26]. The relationship between upregulated ERS/UPR pathways and steroid‐responsiveness in asthma is unknown. However, most cellular and molecular mechanisms described above are related to corticosteroid insensitivity in asthma and linked to ERS, protein misfolding and UPR activation [27, 28, 29].

Here, we extend these findings and show a novel mechanism whereby ERS and the UPR are involved in reduced immunophilin and importins expression and GR nuclear translocation in human cell models of severe TH1/TH17 asthma. We also show how increased ERS/UPR influences the expression of co‐chaperones in GR‐HSP90 heterocomplex, which play a key role in steroid responsiveness. We then demonstrate this can be reversed through treatment with the chemical chaperone, 4‐PBA, that overcomes ERS and enhances corticosteroid responses in human AECs. Finally, we show that 4‐PBA restores responses to corticosteroid treatment in two murine models of severe, corticosteroid‐resistant asthma.

## 2 Methods

### 2.1 Cell Culture

#### 2.1.1 BCi‐NS1.1 Cell Culture

Normal airway epithelium‐derived basal cell line BCi‐NS1.1 cells [30, 31] were cultured in Pneumocult Ex‐plus media (Stemcell technologies) and maintained in T25 cell culture flasks. For stimulations, cells were seeded in 24‐well cell culture plates (1 × 10^5^ cells/well) and used at 80% confluency.

#### 2.1.2 Air–Liquid Interface (ALI) Cultures

pBECs were obtained from non‐disease subjects bronchoscopically [32]. The study protocol was approved by the Hunter New England Human Research Ethics Committee (05/08/10/3.09). All subjects gave informed written consent. pBECs were grown in Pneumocult Ex‐plus media (Stemcell technologies) in submerged monolayer culture first and then seeded at 2 × 10^5^ cells in transwells in a 12‐well plate (Corning) with Pneumocult Ex‐plus media (Stemcell technologies) until confluent (at least 3 days in both apical and basal compartments). Once confluent, the apical media was removed, and the basal media was changed to pneumocult ALI media (Stemcell technologies) and cultured at air‐liquid interface for 21 days until fully differentiated.

#### 2.1.3 Cell Culture Treatments

To mimic TH1/17 cytokine‐induced corticosteroid‐insensitive airways, Bci‐NS1.1 cells or pBEC ALIs were chronically treated with 10 ng/mL IL‐17A, 5 ng/mL IFN‐γ and 1 ng/mL TNF. For Bci‐NS1.1 cells, the cytokine mix was added to submerged cultures for 48 h. For ALI cultures, the cytokine mix was basally added for 10 days. ERS was chemically induced in cell cultures by adding tunicamycin (2 μg/mL, Merk Millipore, USA) or thapsigargin (1 μM, Merk Millipore, USA) into the media for the indicated time points [33]. Dexamethasone (100 nM) was used to treat Bci‐NS1.1 cells or pBEC ALIs for 6–12 h to induce glucocorticosteroid signalling [34, 35]. 4‐PBA (2 mM) was used to treat ERS for the indicated time points. The concentration and the duration of the treatments were determined based on our preliminary data and available literature [33].

### 2.2 Annexin V Cell Viability and Apoptosis Assay

To evaluate if chemically induced ERS leads to cell apoptosis and necrosis, a flow cytometry‐based Annexin V assay was performed on BCi‐NS1.1 cells first challenged with tunicamycin (2 μg/mL) or thapsigargin (1 μM) for 1 h and subsequently cultured for either 2 h or 12 h. Cells treated with ERS inducers for longer durations (48 h) and 0.1 mM Hydrogen Peroxide (H_2_O_2_) for 12 h were used as a positive control for the experiment.

### 2.3 mRNA Extraction and qPCR

Total mRNA was extracted from cells lysed in 350 μL of RLT buffer using RNeasy mini kits (Qiagen, Germany) following the manufacturer's instructions and quantified using a nano‐drop 2000 spectrophotometer (ThermoFisher Scientific). mRNA (200 ng) from each sample was used to produce cDNA with high‐capacity cDNA reverse transcription kits (Applied Biosystems). Genes of interest were quantified by qPCR using TaqMan gene expression assays (ThermoFisher Scientific, Australia) and normalised to the 18S housekeeping gene. Results were calculated using 2‐ΔΔCt (where Ct is the threshold cycle) relative to the mean ΔCt of the healthy control group as described previously [36].

### 2.4 GR Nuclear Translocation Assay by Immunofluorescence Staining

pBECs were treated as above, and after specific time points, cell culture wells were fixed with 10% NBF for 2 h and immunofluorescence staining was performed. Briefly, after fixing, cells were washed with PBS before being permeabilised in 0.5% v/v Triton X‐100 in PBS for 10 min. Non‐specific antibody binding was blocked by the addition of 10% v/v goat serum in PBS for 1 h at room temperature. Cells were then incubated with primary antibody for GR (Abcam ab2768, mouse, diluted 1:250) at 4°C overnight, followed by incubation with the appropriate Alexa Fluor 488 or 555 anti‐mouse IgG conjugates (Cell Signalling Technology) for 1 h at room temperature. Hoechst 33258 (ab228550) was used to stain the nucleus. Images were visualised using an Axio Imager 2 (Zeiss, Oberkochen, Baden‐Württemberg, Germany). Quantitation was achieved using Fiji software (NIH) macros with specific plugins to specifically measure the percentage area of the nucleus associated with fluorescence.

### 2.5 GR Nuclear Translocation by Immunoblotting

Briefly, treated BCi‐NS1.1 cells were harvested and separated into cytoplasmic and nuclear protein fractions using the NE‐PER Nuclear and Cytoplasmic Extraction Reagents kit (ThermoFisher Scientific) according to the manufacturer's guidelines. Protein concentrations were quantified, and equivalent amounts of cytoplasmic and nuclear extracts were subjected to SDS–PAGE followed by immunoblotting as described before [37]. GR protein levels were detected using anti‐GR antibody (Abcam ab2768, mouse, diluted 1:1000). β‐actin and histone H3 were used as the loading control. Protein bands were detected using SuperSignal West Femto Maximum Sensitivity Substrate reagents (ThermoFisher Scientific Inc.) on the ChemiDoc MP Imaging System (Bio‐Rad Laboratories).

### 2.6 Mouse Model of Infection‐Induced Severe Corticosteroid‐Resistant Allergic Airway Disease

To evaluate our results preclinically, murine models of severe, corticosteroid‐resistant (SSR) asthma were utilised as previously described [24, 38]. Animal studies were approved by the University of Newcastle Animal Care and Ethics Committee. Specific pathogen‐free, 6–8‐week‐old, female BALB/c mice were sensitised to ovalbumin (Ova) intraperitoneally (50 μg, Sigma‐Aldrich, Sydney, Australia) in conjunction with the potent, TH2 immune response‐inducing adjuvant aluminium hydroxide (Alhydrogel; 260 μg, InvivoGen, San Diego, California, USA) in sterile saline (200 μL) or sham sensitised with adjuvant alone. Mice were subsequently intranasally challenged with Ova (10 μg, 50 μL PBS) on days 12 and 13 to induce allergic airway disease (AAD), then re‐challenged with Ova on days 33 and 34 to represent an exacerbation of the established disease. AAD was assessed on Day 35 (24 h post final challenge).

To induce SSR disease, AAD is first established, and then mice were intranasally inoculated on Day 14 with either *Chlamydia muridarum* (*Cmu*; 100 IFU, ATCC VR‐123, in 30 μL sucrose phosphate glutamate [SPG] buffer) or non‐typeable *Haemophilus influenzae* (Hinf; 8x10^6^ CFU, NTHi‐289, in PBS) or sham inoculated with SPG (sham infection for Cmu) or PBS (sham infection for Hinf) [24, 38]. To treat disease, mice were intranasally treated on days 32–34 with dexamethasone (Dex; 2 mg/kg in PBS; Sigma‐Aldrich) alone or in combination with 4‐PBA (8 mg/kg). Timelines for each treatment for two different mouse models are displayed as supplementary data.

### 2.7 Lung Function Analyses

Mice were anesthetised with ketamine (100 mg/kg) and xylazine (10 mg/kg), and tracheas were cannulated (tracheostomy with ligation) [24, 38]. FlexiVent apparatus (FX1 System; SCIREQ, Montreal, Canada) was used to assess airway‐specific resistance (Rn; tidal volume of 8 mL/kg at a respiratory rate of 450 breaths/min) in response to increasing doses of nebulized methacholine (Sigma‐Aldrich). Assessment of the effects of each dose of saline/methacholine on lung function parameters was performed at least three times, and the average was calculated. AHR is expressed as a percentage change from baseline (nebulised saline) responses.

### 2.8 Airway Inflammation

Airways of mice were lavaged twice with 1 mL of Hank's buffered salt solution (HBSS; Sigma‐Aldrich) via a cannula inserted into the trachea. Bronchoalveolar lavage fluid was centrifuged (300 × *g*, 10 min, 4°C), red blood cells lysed (200 μL, Tris‐buffered NH_4_Cl) and remaining cells pelleted before total leukocyte numbers were determined using a haemocytometer and trypan blue exclusion. Cells were cytocentrifuged and stained with May–Grunwald–Giemsa. Differential cell counts were determined by morphology under light microscopy (40× magnification) [24, 38].

### 2.9 Sputum Gene Correlation Analysis

For sputum samples, we extracted gene expression data from our existing microarray database (GSE137268), consisting of participants with severe asthma (*n* = 13, Table 1) [39]. The correlations between important ERS/UPR gene expressions, the components of GR‐HSP90 heterocomplex, steroid‐responsive genes, and the asthma six‐gene signature genes were analysed.

**TABLE 1:** Clinical characteristics and inflammatory cell profiles of participants with severe asthma involved in the sputum microarray study.

	Severe asthma
Participants (*n*)	13
Age (years) (median, IQR)	64 (53–68)
Sex *n* (%)	Male 4 (30%), female 9 (70%)
Atopy (*n*/%)	10 (76)
FEV1% predicted (median, IQR)	63 (53–73)
FEV1/FVC % (median, IQR)	62 (56–71)
ICS dose (mcg Budesonide dose/day) median, IQR	1600 (1000–2000)
ACQ (median, IQR)	1.35 (0.61–1.93)
TCC ×10^6^ (median, IQR)	3.42 (2.25–6.39)
Viability (median, IQR)	89.47 (71.05–91.46)
Neutrophils % median, IQR	66.5 (39.25–75.25)
Eosinophils % median, IQR	3 (0.75–24.75)
Macrophages % median, IQR	22.6 (8.5–28.25)

*Note:* Features of participants with asthma were further categorised based on clinical severity following GINA guidelines.

Abbreviations: ACQ, Asthma Control Questionnaire; FEV1, forced expiratory volume in 1 s; FVC, forced vital capacity; ICS, Inhaled corticosteroid; TCC, Total cell count.

### 2.10 Statistical Analysis

Data was analysed using GraphPad Prism 9.0.0 software. Multiple comparisons between groups were analysed with an appropriate test for normally distributed data with Analysis of Variance (ANOVA), followed by post hoc analysis. Two‐group comparisons were analysed with an unpaired *t*‐test. Correlation analyses were performed using Spearman's rank test. *p* values from the multiple correlation analyses were adjusted using stacked *p*‐value analysis in GraphPad Prism, applying a 5% False Discovery Rate (FDR) correction to control the Type I error rate associated with multiple comparisons. Individual two‐gene correlations were not corrected for multiple testing and are intended to be exploratory. Airways hyperresponsiveness (AHR) data were assessed using a two‐way analysis of variance with a post hoc test. Statistical significance of *p* < 0.05 was accepted with a 95% confidence interval.

## 3 Results

### 3.1 ERS Impairs Glucocorticoid‐Responsive Genes, GR‐Associated Importins and GR Nuclear Translocation in Human Airway Epithelial Cells

**FIGURE 1:**
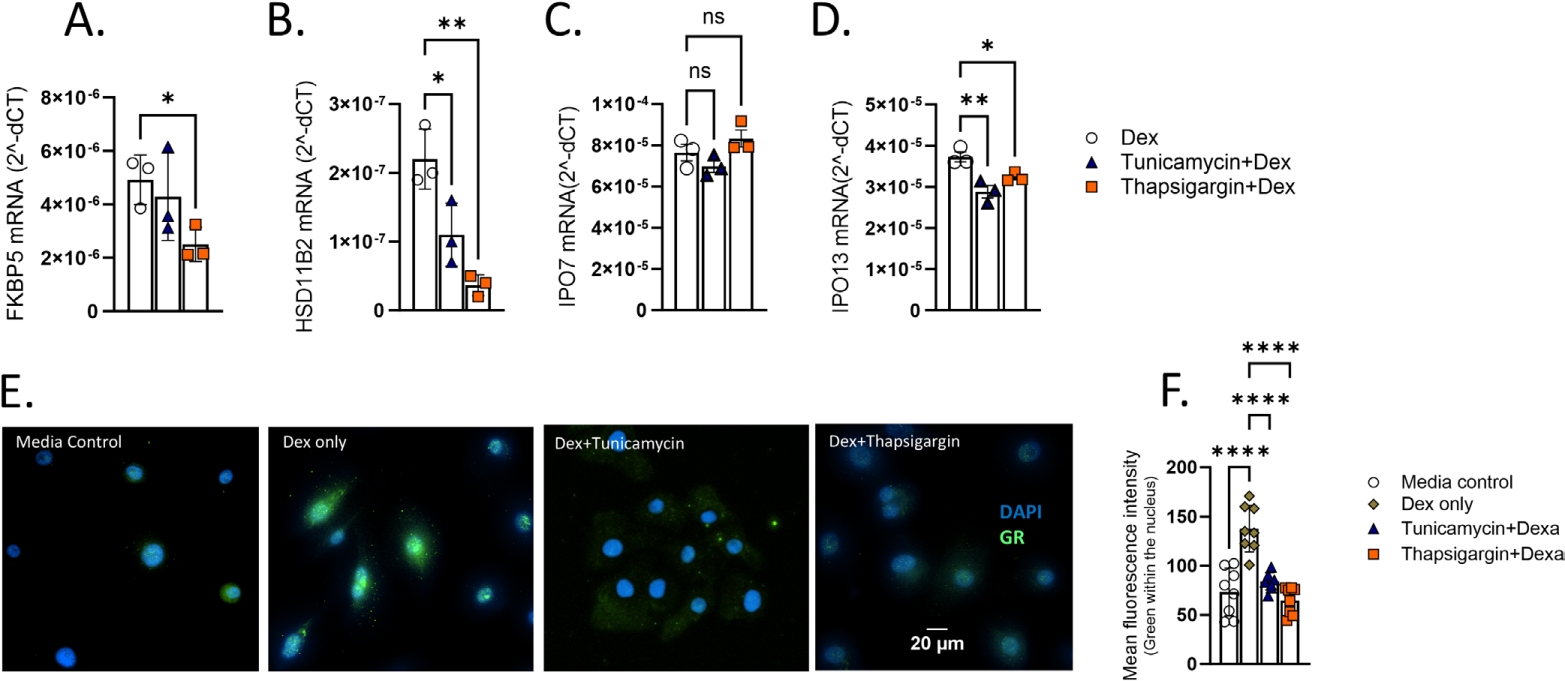
ER stress impairs glucocorticoid responsiveness in airway epithelial cells. Bci‐NS1.1 cells (*n* = 3) were first challenged with tunicamycin (2 μg/mL) or thapsigargin (1 μM) for 1 h. Cultures were then replenished with media containing dexamethasone (Dex, 100 nM) and cultured for 12 h. Gene induction of glucocorticoid‐responsive genes, (A) *FKBP5* and (B) *HSD11B2* and GR‐associated importins, (C) *IPO7* (D) *IPO13* was measured by qPCR. To assess GR nuclear translocation, Bci‐NS1.1 cells (*n* = 8) were challenged with either tunicamycin (2 μg/mL) or thapsigargin (1 μM) for 1 h and then treated with Dex (100 nM) for 2 h. Cells were fixed, and immunofluorescence was performed. (E) Representative single‐plane 2D images show GR (green colour) and nuclear (blue colour) translocation. (F) Mean fluorescence intensity was measured using ImageJ software. *p < 0.05, **p < 0.01, ****p < 0.0001. “*n*” denotes the number of independent experiments, with each experiment including two technical replicates that were combined and expressed as the mean for subsequent analysis.

To demonstrate if ERS impairs the glucocorticoid signalling pathway in human airway epithelial cells, Bci‐NS1.1 cells were first challenged with tunicamycin (2 μg/mL) or thapsigargin (1 μM) for 1 h and subsequently cultured for 12 h in the presence of 100 nM of dexamethasone in cultures. After 12 h of treatment, cells were harvested, and the expression of glucocorticoid‐responsive genes, FKBP5 and HSD11B2, was measured by qPCR. Gene expression of both FKBP5 and HSD11B2 was substantially downregulated by thapsigargin, whilst tunicamycin only reduced HSD11B2 expression (Figure 1A,B). To further evaluate if ERS has a direct impact on GR‐associated importins, the gene expression of importin 7 and 13 (IPO7 and 13) was measured. Treatment with both tunicamycin and thapsigargin significantly reduced the expression of IPO13 (Figure 1D), with no effect on IPO7 (Figure 1C).

To assess the level of GR nuclear translocation, Bci‐NS1.1 cells were challenged with thapsigargin or tunicamycin for 1 h and then treated with 100 nM dexamethasone for 2 h. GR nuclear translocation was measured at 2 h time points using immunofluorescence staining. Both thapsigargin and tunicamycin reduced dex induced GR nuclear translocation, while the effect of thapsigargin was more pronounced (Figure 1E,F).

To evaluate if our treatment conditions induce apoptosis and cell death, and skew the results, each condition was evaluated for early‐late apoptotic and necrotic events using the Annexin V apoptosis detection kit. BCi‐NS1.1 cells treated with ERS inducers for a longer period (48 h) or with 0.1 mM H_2_O_2_ for 12 h were used as positive controls. Treatment with both tunicamycin (2 μg/mL) or thapsigargin (1 μM) for 1 h and subsequently cultured for either 2 or 12 h did not significantly induce apoptosis or necrosis in BCi‐NS1.1 cells. Cells retaining 95% viability, indicating that cell death did not influence these results (Figure S1).

### 3.2 ERS and UPR Signalling Gene Expression Modulate the Expression of Components of the GR‐HSP90 Heterocomplex and Steroid‐Responsive Genes

**FIGURE 2:**
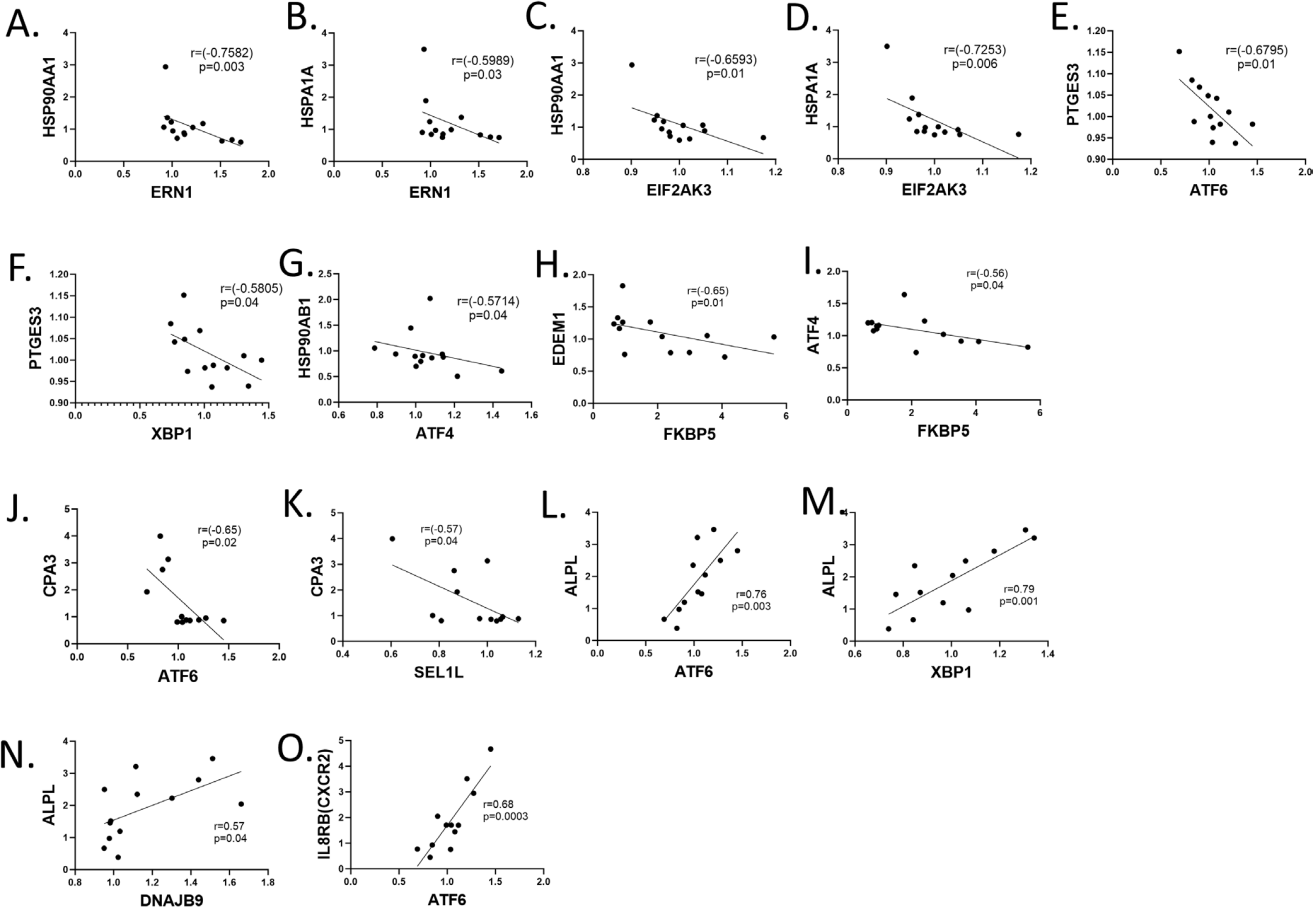
ERS/UPR gene expression correlates with the co‐chaperones of GR‐HSP90 heterocomplex, GR‐associated immunophilins, and asthma signature genes. Gene expression data from microarray analysis of severe asthma sputum (*n* = 13, where “*n*” corresponds to the number of individual patient samples) were assessed for correlations between ERS/UPR (ERN1, EIF2AK3, ATF6, XBP1, ATF4, EDEM1, SEL1L, DNAJB9) and GR‐HSP90 heterocomplex components (HSP90AA1, HSPA1A, PTGES3, HSP90AB1), ERS/UPR and GR‐immunophilins (FKBP5), ERS/UPR and asthma signature genes (CPA3, ALPL, CXCR2) using Spearman's rank correlation test. The correlation matrix with correlation coefficients is displayed in Supporting Information (Figure S2). The gene expression of *ERN1* negatively correlates with (A) *HSP90AA1* and (B) *HSPA1A*. The gene expression of *EIF2AK3* negatively correlates with (C) *HSP90AA1* and (D) *HSPA1A*. The gene expression of (E) *ATF6* and (F) *XBP1* negatively correlates with *PTGES3*. (G) The gene expression of *ATF4* negatively correlates with *HSP90AB1*. GR‐associated immunophilin FKBP5 negatively correlates with (H) EDEM1 and (I) ATF4. A marker of eosinophilic asthma, CPA3, negatively correlates with (J) ATF6 and (K) SEL1L. A marker of neutrophilic asthma, ALPL, positively correlates with (L) ATF6 (M) XBP1 and (N) DNAJB9. (O) A marker of neutrophilic asthma, CXCR2, positively correlates with ATF6. Correlation plots of selected genes were identified from the correlation matrix. While the FDR correction was applied to the matrix analysis, individual two‐gene correlations were not corrected for multiple testing and are intended to be exploratory.

To investigate if ERS impacts the expression of components of the GR‐HSP90 heterocomplex in sputum cells of severe asthma, a correlation analysis was performed. Patient demographics are displayed in Table 1. The correlation between the gene expression of HSP90 (*HSP90AA1*, *HSP90AB1*), P23 (*PTGES3*), HSP70 (*HSPA1A*) was compared with ERS and UPR genes; PERK (*EIF2AK3*), IRE1α (*ERN1*), *ATF6*, *ATF4, XBP1*, *EDEM1*, BIP (*HSPA5*), and CHOP (*DDIT3*) and displayed as a correlation matrix (Figure S2). Genes with a significant correlation were individually graphed (Figure 2A–G). Our results show that, ERS/UPR gene *ERN1* and *EIF2 AK3* negatively correlate with *HSP90AA1* and *HSPA1A* (Figure 2A–D), *ATF6* and *XBP1* negatively correlate with P23 (*PTGES3*) (Figure 2E,F), and *ATF4* negatively correlates with *HSP90AB1* (Figure 2G). We also investigate the correlation between ERS/UPR gene and steroid‐responsive genes FKBP5 (immunophilin) and HSD11B2 (Figure S2B). Consistent with our findings in vitro, ERS/UPR genes, EDEM1 and ATF4, negatively correlate with immunophilin, FKBP5 (Figure 2H,I). These results indicate that increased ERS/UPR gene expression in lung immune cells of severe asthma patients leads to reduced expression of components of GR‐HSP90 heterocomplex, resulting in impaired GR signalling.

The relationship between the ERS/UPR genes and the components of GR‐HSP90 heterocomplex based on disease severity and phenotype was further analysed in an independent dataset from the U‐BIOPRED study and displayed as Supporting Information (Figure S3).

### 3.3 ERS/UPR Gene Expression Correlates With the Asthma Six‐Gene Signature

Asthma six‐gene signature developed by Baines et al. is an important molecular diagnostic tool for identifying inflammatory phenotypes of asthma and predicting patient response to inhaled corticosteroids. This signature, which includes genes CLC, CPA3, DNASE1L3, IL1B, ALPL and CXCR2, offers improved performance over traditional measures in predicting treatment response and the risk of future severe exacerbations, potentially guiding personalised asthma management. Our results demonstrate that ERS/UPR genes ATF6 and SEL1L negatively correlate with CPA3, a marker of eosinophilic asthma (Figure 2J,K), which is expected to be high in steroid responders. Conversely, ATF6, XBP1 and DNAJB9 show a positive correlation with ALPL and CXCR2, markers associated with steroid‐insensitive severe neutrophilic asthma (Figure 2L–O and Figure 2C). These results further confirm that certain ERS/UPR signalling pathways are upregulated in steroid‐insensitive asthma.

### 3.4 TH 1/17 Cytokine Mix Induces Corticosteroid Insensitivity and ERS in Airway Epithelial Cells

**FIGURE 3:**
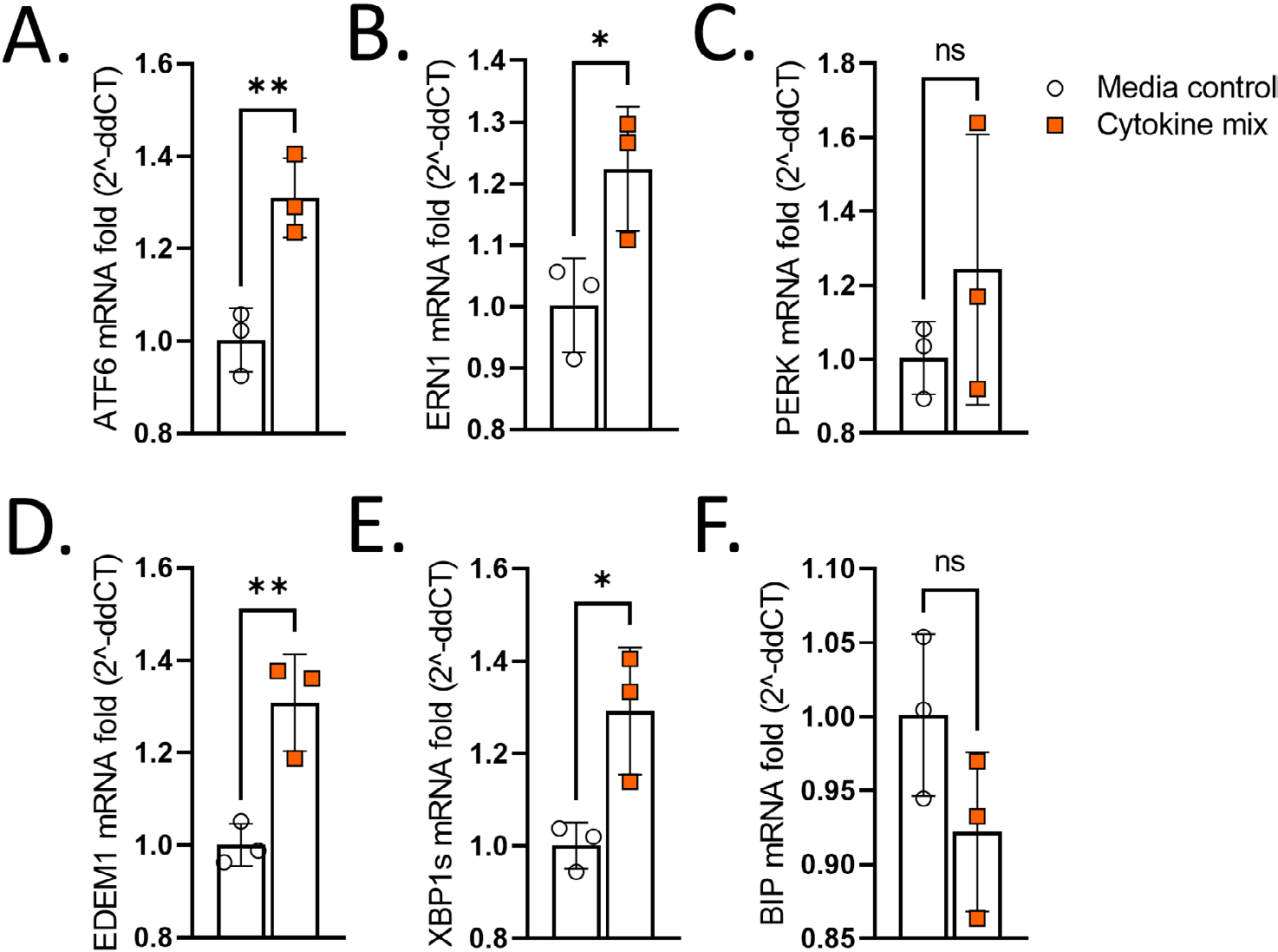
TH1/17 cytokine mix induces ER stress in airway epithelial cells. BCi‐NS1.1 cells (*n* = 3) were treated with TH1/17 cytokine mix for 48 h and the gene expression of (A). *ATF6* (B). *ERN1* (C). PERK (*EIF2AK3*) (D). *EDEM1* (E). *XBP1s* (F). BiP (*HSPA5*) was measured by qPCR. *p < 0.05, **p < 0.01. “*n*” denotes the number of independent experiments, with each experiment including two technical replicates that were combined and expressed as the mean for subsequent analysis.

TH 1/17 cytokines have been shown to induce corticosteroid insensitivity in airway cells [34, 40, 41]. Whether these cytokines induce ERS and UPR in airway epithelial cells has not been defined. We first investigated if TH1/17 cytokine mix that we use in this study (10 ng/mL IL‐17A, 5 ng/mL IFN‐ɣ and 1 ng/mL TNF) induces the ERS/UPR gene expression in Bci‐NS1.1 cells. TH1/17 cytokine mix induced the gene expression of UPR transducers; ATF6 and ERN1 (Figure 3A,B), misfolded protein marker; EDEM1 (Figure 3D), and ERS‐induced transcription factor; XBP1s (Figure 3E) confirming that neutrophils inflammation enhance ERS/UPR in airway epithelium.

### 3.5 Chemical Chaperone 4‐PBA Restores Corticosteroid Sensitivity in Airway Epithelial Cells

**FIGURE 4:**
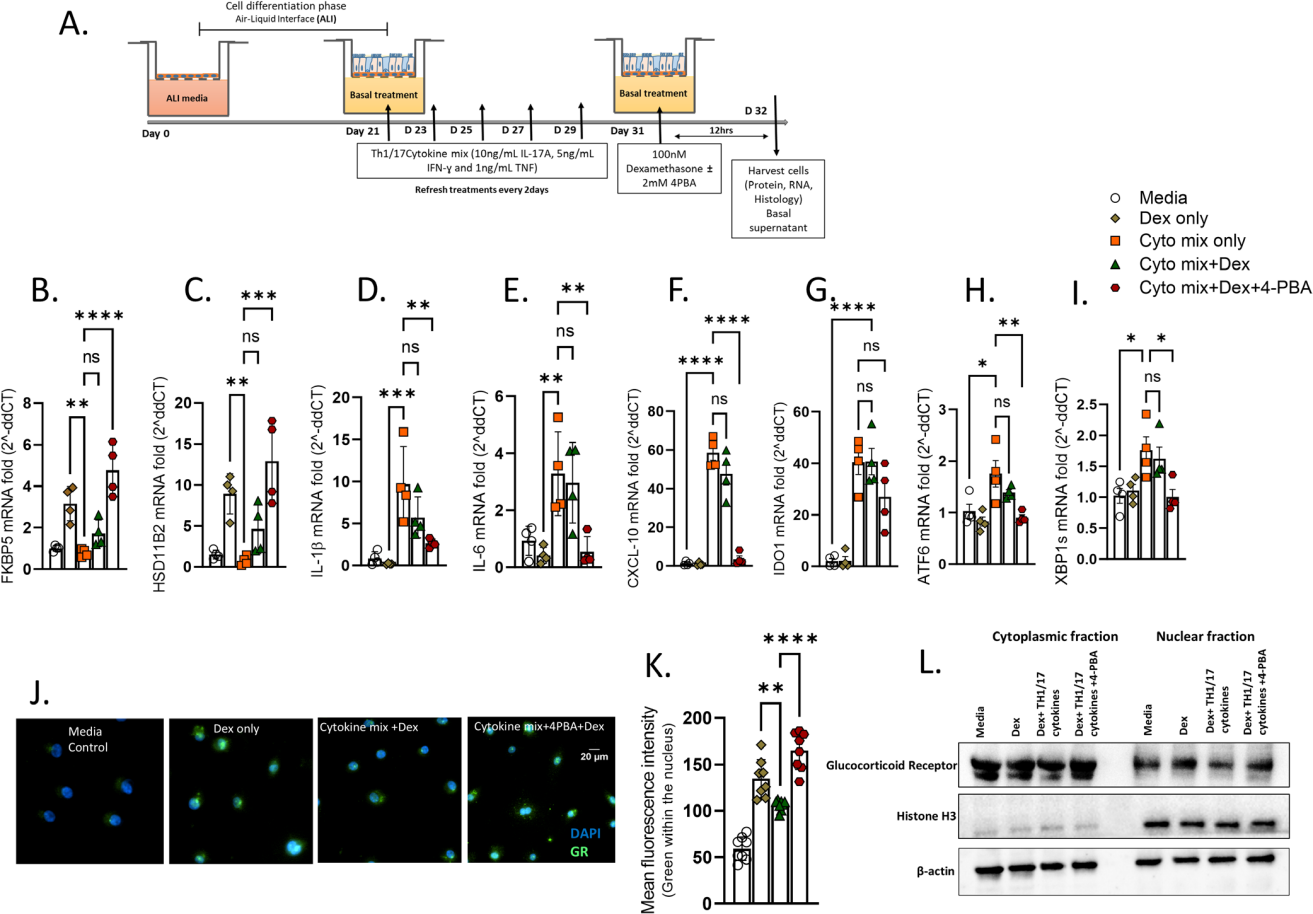
Treatment with 4‐PBA enhances corticosteroid responses in airway epithelial cells. (A) A schematic diagram shows the process of how primary broncho‐epithelial cells (pBECs, *n* = 4, where “*n*” denotes the number of individual patient‐derived biological samples (independent donors). Each sample was analysed in two technical replicates, which were pooled and expressed as the mean for subsequent analysis.) differentiated at the air‐liquid interface (ALI) were challenged with TH1/17 cytokine mix containing 10 ng/mL IL‐17A, 5 ng/mL IFN‐ɣ and 1 ng/mL TNF for 10 days to induce corticosteroid insensitivity. The basal media containing the cytokine mix was replenished every 2 days. At the end of the cytokine treatment, ALIs were treated with dexamethasone (Dex, 100 nM) alone or with 4‐PBA (2 mM) for 12 h. Corticosteroid‐responsive genes (B) FKBP5 (C) HSD11B2, inflammatory genes (D) IL‐1β (E) IL‐6, Interferon regulatory genes (F) CXCL10 (G) IDO1 and ER stress/UPR genes (H) ATF6 (I) XBP1 were measured by qPCR. To assess GR nuclear translocation, Bci‐NS1.1 cells (*n* = 8, where “*n*” denotes the number of independent experiments, with each experiment including two technical replicates that were combined and expressed as the mean for subsequent analysis) were challenged with TH1/17 cytokine mix for 48 h and then treated with Dex (100 nM) alone or with 2 mM 4‐PBA for 2 h. Cells were fixed, and immunofluorescence was performed. (J) Representative single‐plane 2D images show GR (green colour) nuclear (blue colour) translocation. (K) Mean fluorescence intensity was measured using ImageJ software. (L) Immunoblot analysis shows GR protein expression in BCi‐NS1.1 cells following the indicated treatments, in both the cytoplasmic and nuclear fractions. *p < 0.05, **p < 0.01, ***p < 0.001, ****p < 0.0001.

We then sought to demonstrate whether resolving ER protein misfolding by treating with a chemical chaperone, 4‐PBA, can improve TH1/17 cytokine‐induced corticosteroid insensitivity in airway epithelial cells. To assess this, we challenged both basal epithelial cells (Bci‐Ns1.1 cells) and differentiated pBECs with TH1/17 cytokine mix to induce corticosteroid insensitivity and then treated with dexamethasone with or without 4‐PBA. In this study, corticosteroid insensitivity was operationally defined as an attenuated response to dexamethasone, characterised by reduced induction of glucocorticoid‐responsive genes (FKBP5, HSD11B2) and diminished glucocorticoid receptor (GR) nuclear translocation under cytokine‐exposed conditions, relative to dexamethasone‐treated controls. Treatment of pBECs with 4‐PBA in combination with dexamethasone increased the expression of dexamethasone‐responsive genes while dexamethasone alone was ineffective (Figure 4B,C). We also measured the expression of proinflammatory genes induced by TH1/17 cytokines. TH1/17 cytokine challenge substantially upregulated IL‐6 and IL‐1β gene expression, and treatment with dexamethasone together with 4‐PBA significantly reduced their expression (Figure 4D,E). We further investigated how interferon stimulatory genes (ISGs) are expressed in our model and the efficacy of 4‐PBA in controlling interferon‐stimulated inflammation in cells. TH1/17 cytokine mix significantly induced CXCL‐10 and IDO1 gene expression, and treatment with 4‐PBA with dexamethasone effectively resolved interferon‐stimulated inflammatory responses (Figure 4F,G) reflecting that 4‐PBA could be a potential treatment for virus infection‐induced steroid resistance in asthma [31]. In addition, our data shows that certain ERS/UPR signalling pathways are upregulated in our model, and 4‐PBA is effective in downregulating them (Figure 4H,I and Figure S4).

We then investigated if resolving ER protein misfolding using 4‐PBA improves dexamethasone‐induced GR nuclear translocation in basal epithelial cells. Our immunofluorescence data show challenge with TH1/17 cytokine mix reduced total GR expression as well as dexamethasone‐induced GR nuclear translocation and treatment with dexamethasone in combination with 4‐PBA significantly increases the GR nuclear translocation (Figure 4J,K). Further, these results were confirmed by performing immunoblotting for GR protein separately for the cytoplasmic and nuclear fractions (Figure 4L). These results suggest that treatment with 4‐PBA enhances GR activation and nuclear translocation in the airway epithelium.

### 3.6 Resolving ERS by Treatment With Chemical Chaperones Improves Corticosteroid Responsiveness in Two Murine Models of Severe, Corticosteroid‐Insensitive Asthma

**FIGURE 5:**
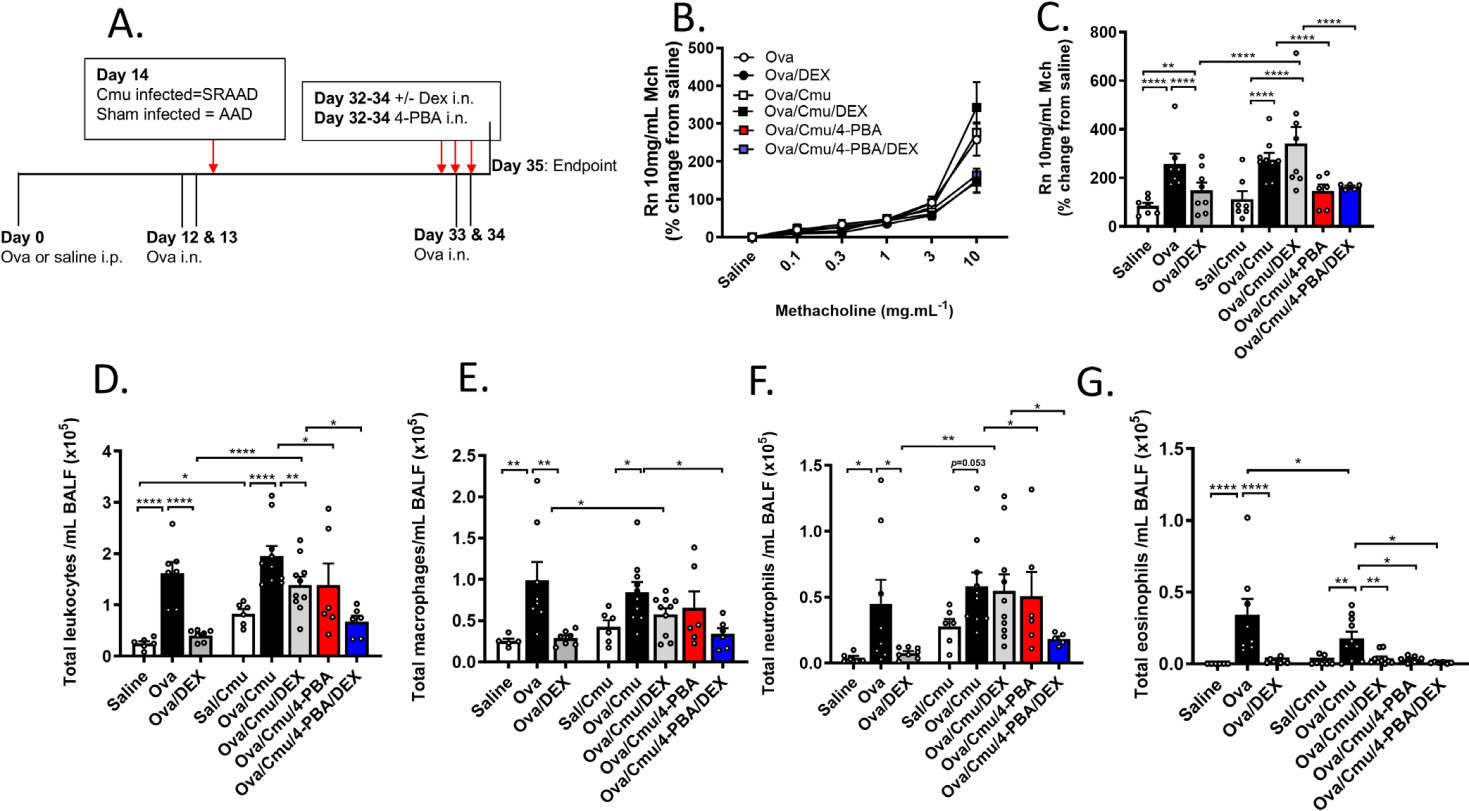
Treatment with 4‐PBA protects against hallmark features of *Chlamydia muridarum* (Cmu)‐induced severe corticosteroid‐resistant allergic airways disease (SRAAD). (A) Mice were sensitised with intraperitoneal (i.p.) injection of ovalbumin and aluminium hydroxide (Ova, Day 0) and subsequently challenged intranasally (i.n.) with Ova to establish AAD (Days 12, 13, 33 and 34). SRAAD was induced by infecting mice i.n. with Cmu (Day 14). The effects of i.n. treatments with dexamethasone (Dex; 2 mg/kg) or 4‐PBA (8 mg/kg) alone or in combination were investigated (Days 32–34). Controls were sham‐sensitised with i.p injection of saline and aluminium hydroxide (Sal, Day 0), sham‐infected with sucrose phosphate glutamate buffer (Day 14) and/or sham‐treated with PBS (Days 32–34). (B) At Day 35, airway hyper‐responsivity was assessed by measurement of airway resistance (Rn) in response to increasing doses of methacholine (Mch) with data represented as Rn for the dose–response curve and (C) at the maximal Mch dose (10 mg/mL). At Day 35 (D) Total leukocyte, (E) macrophage, (F) neutrophil and (G) eosinophil numbers in bronchoalveolar lavage fluid (BALF) were enumerated. Data represented as mean ± SEM. *p < 0.05, **p < 0.01, and ****p < 0.0001 denotes statistical differences between groups. Individual biological replicates, denoted by individual circles within the histograms, were collected across two or more independent experimental endpoint days with mouse numbers in each group distributed across multiple endpoint days (typically 2–5 mice per endpoint) to achieve the final sample size reported in the figures (*n* = 5–10). The timeline for each treatment is displayed in the Supporting Information (Figure S7).

**FIGURE 6:**
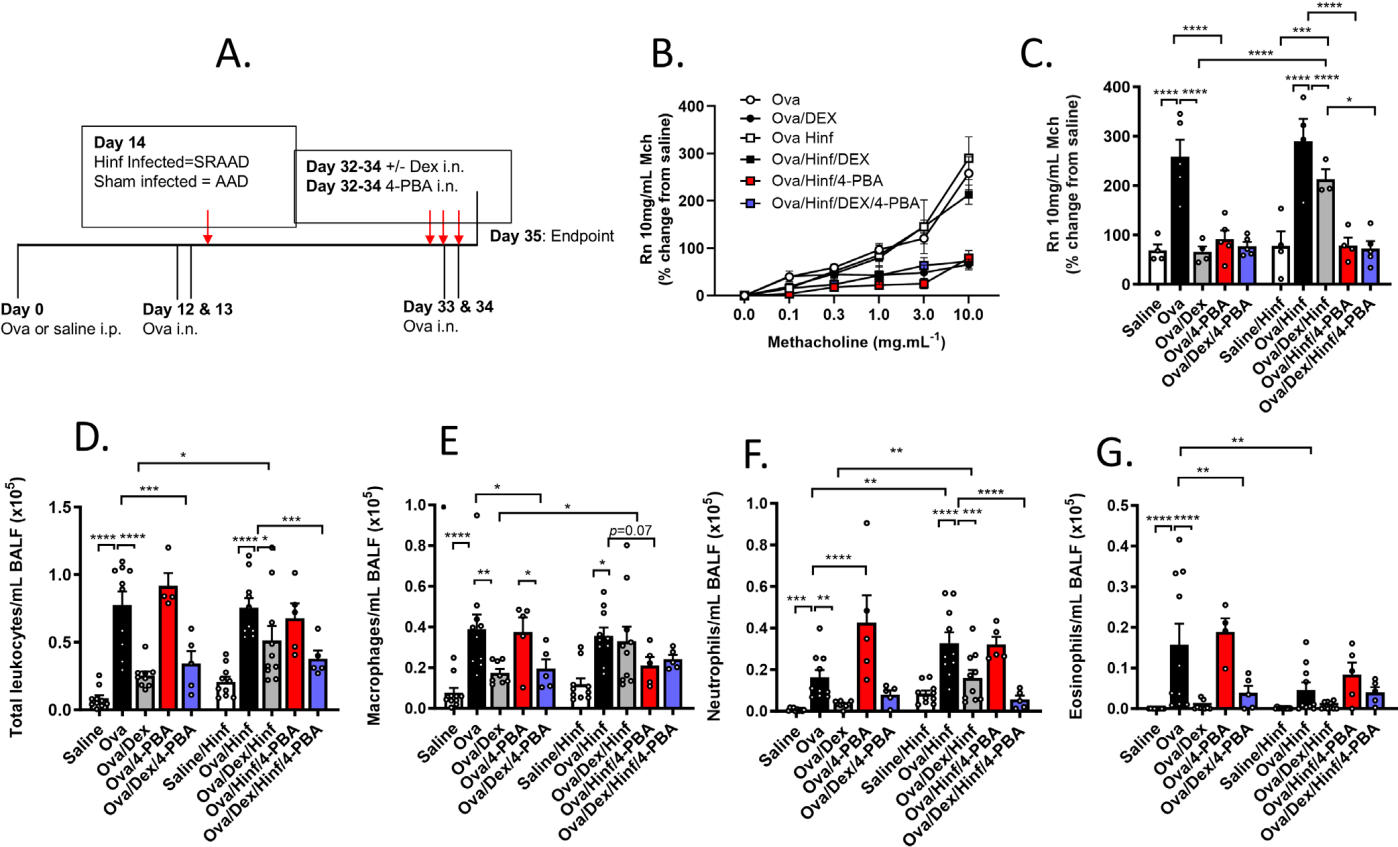
Treatment with 4‐PBA protects against hallmark features of non‐typeable *Haemophilus influenzae* (Hinf)‐induced severe corticosteroid‐resistant allergic airways disease (SRAAD). (A) Mice were sensitised with intraperitoneal (i.p.) injection of ovalbumin and aluminium hydroxide (Ova, Day 0) and subsequently challenged intranasally (i.n.) with Ova to establish AAD (Days 12, 13, 33 and 34). SRAAD was induced by infecting mice i.n. with Hinf (Day 14). The effects of i.n. treatments with dexamethasone (Dex; 2 mg/kg) or 4‐PBA (8 mg/kg) alone or in combination were investigated (Days 32–34). Controls were sham‐sensitised with i.p injection of saline and aluminium hydroxide (Sal, Day 0), sham‐infected with PBS (Day 14) and/or sham‐treated with PBS (Days 32–34). At Day 35 airways hyper‐responsiveness was assessed by measurement of airways resistance (Rn) (B) in response to increasing doses of methacholine (Mch), with data represented as Rn for the dose–response curve and (C) at the maximal Mch dose (10 mg/mL). At Day 35 (D) Total leukocyte, (E) macrophage, (F) neutrophil and (G) eosinophil numbers in bronchoalveolar lavage fluid (BALF) were enumerated. Data represented as mean ± SEM. *p < 0.05, **p < 0.01, ***p < 0.001 and ****p < 0.0001 denotes statistical differences between groups. Individual biological replicates, denoted by individual circles within the histograms, were collected across two or more independent experimental endpoint days with mouse numbers in each group distributed across multiple endpoint days (typically 2–5 mice per endpoint) to achieve the final sample size reported in the figures (*n* = 4–10). The timeline for each treatment is displayed in the Supporting Information (Figure S8).

We used two different pre‐clinical models of severe, corticosteroid‐resistant allergic airways disease (SRAAD) to determine whether targeting ER stress in vivo can enhance corticosteroid responsiveness (Figures 5A and 6A) [24, 38, 42]. Both models utilise OVA‐induced allergic airways disease (AAD) that is sensitive to Dex treatment. Corticosteroid (Dex)‐insensitive inflammation and AHR are induced in this model of OVA‐induced AAD by infecting mice with Cmu or Hinf, which promote corticosteroid‐insensitivity through the induction of TH1/TH17 responses during OVA‐induced AAD [24, 38, 43]. The effects of 4‐PBA treatment, in the presence and absence of Dex treatment, on AHR and airway inflammation were assessed in both Cmu‐ and Hinf‐induced SRAAD. We show that Dex treatment suppresses both AHR and immune cell numbers in bronchoalveolar lavage fluid (BALF) of mice with OVA‐induced AAD without infection (Figures 5 and 6). Both Cmu and Hinf infections result in AHR and total leukocyte, macrophage and/or neutrophil numbers in BALF that are not suppressed, or only partially suppressed, by Dex treatment (Figures 5 and 6). 4‐PBA effectively suppresses AHR in OVA‐induced AAD and, unlike Dex, suppresses AHR in Cmu‐ and Hinf‐induced SRAAD (Figures 5C and 6C). Indeed, 4‐PBA suppresses AHR in corticosteroid‐sensitive AAD as well as Cmu‐ and Hinf‐induced SRAAD, even when not combined with Dex treatment. Interestingly, we show that 4‐PBA treatment alone has little effect on inflammatory cell numbers in either Ova‐induced AAD or in Cmu‐ or Hinf‐induced SRAAD, but it does reduce corticosteroid‐resistant total leukocyte, macrophage and/or neutrophil numbers in BALF in Cmu‐ and/or Hinf‐induced SRAAD when combined with Dex. These data indicate that 4‐PBA is at least as if not more effective than corticosteroids at suppressing AHR and that 4‐PBA improves corticosteroid responses in suppressing airways inflammation in our murine models of corticosteroid‐insensitive and severe asthma.

## 4 Discussion

People with severe asthma experience persistent symptoms, frequent asthma exacerbations, and progressive loss of lung function. While the proportion of people with severe asthma is only ~10%, they experience frequent healthcare visits and account for significant medical costs, 50%–80% higher than those with stable asthma [44]. They are also exposed to frequent courses of oral corticosteroids, leading to severe adverse effects and suffer a reduced quality of life [45]. Monoclonal antibody therapies have emerged in the last 15 years that effectively reduce exacerbations and systemic corticosteroid use [46]. However, these agents are highly specific and only beneficial for people with severe allergic and eosinophilic asthma with T‐helper type‐2 (TH2) mediated inflammation. They are not efficacious in severe asthma associated with neutrophilic inflammation mediated by TH1‐ and TH17‐driven inflammation [47]. Monoclonal antibody therapy is also expensive, and treatment once begun is likely needed for the rest of a patient's life [48, 49]. Even in those who are clinical responders, < 1 in 5 achieve clinical remission, leaving the majority with a significant ongoing symptom burden [50]. Restoring inhaled corticosteroid sensitivity would provide a strategy that could modify the disease process and return people with severe asthma to a less severe disease course.

ERS and protein misfolding are known to be associated with asthma. Previously, we and others showed increased ERS/UPR markers in clinical samples from patients with asthma and that these were associated with greater disease severity [25, 51]. Some studies of experimental models of asthma speculated that ERS/UPR pathways could lead to corticosteroid resistance in severe asthma [52]. These pathways include JNK‐AP‐1 activation via IRE1α [51, 53, 54], NF‐κb activation via IRE1α or ATF6 [55, 56] and PI3K activation via XBP1 or ATF6 [57, 58]. However, none of these studies showed a direct effect of ERS or protein misfolding on GR nuclear translocation or corticosteroid‐responsive gene expression. This study, for the first time, shows that ERS directly induces corticosteroid insensitivity in human airway epithelial cells and, intriguingly, facilitates ER protein folding capacity using an FDA‐approved chemical chaperone, 4‐PBA, which reinstates corticosteroid responsiveness. 4‐PBA is FDA‐approved and is currently used as an oral preparation to treat urea cycle disorders with minimal side effects [59, 60, 61]. Its utility at the moment is limited by cost, but our results demonstrate an important proof of concept of its effectiveness in reversing corticosteroid sensitivity. Future work should determine if these effects are long‐lasting or require repeat administration. Delivery of 4‐PBA could be either systemic or inhaled, ideally coupled with the administration of inhaled corticosteroids.

Our results show that ERS directly reduces GR nuclear translocation and reduces dexamethasone‐responsive genes (FKBP5, HSD11B2) in pBECs. FKBP5 (aka FKBP51) is a chaperone component of the GR‐HSP90 heterocomplex [62] and has been shown to modulate corticosteroid sensitivity [34, 63]. It has also been shown to be upregulated by dexamethasone treatment [34, 63]. HSD11B2 is a NAD^+^ dependent enzyme that is involved in glucocorticoid homeostasis. It is highly upregulated upon dexamethasone exposure in airway epithelial cells [34, 64]. Our results show that ERS induction downregulates dexamethasone‐induced FKBP5 and HSD11B2 expression in airway epithelial cells. Similarly, the mix of TH1/17 cytokines that are shown to be upregulated in the lungs of patients with severe corticosteroid‐resistant asthma also reduced the expression of these genes while upregulating ERS/UPR genes in BECs. These results provide strong evidence that ERS directly interferes with corticosteroid sensitivity and activation in airway epithelial cells. We also observed that ERS inducers significantly downregulate Importin 13 (IPO13) expression in airway epithelial cells. IPO13 is a key nuclear transport receptor that shuttles glucocorticoid receptor (GR) complexes into the nucleus [65], enabling efficient transcription of glucocorticoid‐responsive genes that mediate anti‐inflammatory and barrier‐protective effects. The reduced expression of IPO13 by ERS suggests that cellular stress can uncouple GR activation from its downstream genomic actions. This provides a mechanistic link of how heightened ERS in severe asthma may limit IPO13 availability, restrict GR nuclear translocation and thereby blunt the therapeutic efficacy of corticosteroids.

Treatment with 4‐PBA together with dexamethasone effectively enhances the expression of steroid‐responsive genes, reduces proinflammatory genes, and reduces interferon‐stimulated inflammatory responses in our steroid‐insensitive ALI model, whereas steroids alone are ineffective. These results together suggest potential applicability of 4‐PBA in clinical settings as an add‐on treatment for severe neutrophilic steroid‐insensitive asthma or even in virus‐induced steroid‐insensitivity. While promising, these transcriptional results benefit from confirmation via complementary protein‐level investigations. However, in the current study, we were unable to collect apical secretions for the detection of secreted inflammatory cytokines, as the apical surface of our in vitro model was specifically designed to remain exposed to air. The proinflammatory cytokine levels in basal supernatants were detected below threshold, likely due to preferential apical secretion in ALI cultures and the routine replacement of basal medium every 2 days, which limits protein accumulation. Therefore, future experiments dedicated to investigating the proteomic effects of 4‐PBA on the regulation of inflammatory responses would be important.

Despite the therapeutic effect of 4‐PBA in restoring steroid responses, we observed a mixed response in reducing ERS/UPR‐related genes in both in vitro and in vivo (Figures S4–S6). Given that ERS/UPR constitutes a complex signalling network involving numerous pathways, and that 4‐PBA functions as a pan‐ER‐stress inhibitor with additional HDAC‐inhibitory activity, the precise gene targets affected by 4‐PBA cannot be fully delineated in the current study. Further investigations aimed at dissecting the molecular pathways underlying these effects will be essential for understanding the precise mechanisms of how 4‐PBA enhances steroid responsiveness in cells.

Our correlation analysis in sputum samples of severe asthma shows a strong relationship between the gene expression of the components of GR‐HSP90 heterocomplex and ERS/UPR genes. These results suggest that the expression of the accessory chaperones in the GR‐HSP90 heterocomplex is highly dependent on the status of the protein misfolding/ERS in severe asthma immune cells. We also explored the relationship between ER stress/UPR‐related genes and GR chaperone genes in an independent cohort using data from the U‐BIOPRED severe asthma cohort (Figure S3). Within this cohort, we observed strong correlations between ER stress/UPR markers and GR chaperone genes; however, the pattern and directionality of these associations differed from those observed in our microarray cohort. A key consideration in regards is that our severe asthma microarray cohort represents a distinct patient population characterised by uncontrolled disease despite very high doses of inhaled corticosteroids and markedly impaired lung function. These features differ from the U‐BIOPRED definitions of severe asthma. When we applied additional stratification within the U‐BIOPRED dataset, selecting subjects with poor lung function and specific inflammatory profiles, and further categorising them into Th1‐ and Th2‐skewed subgroups, we observed a correlation pattern more consistent with our findings, particularly within the Th1 subgroup (Figure S3D). However, the small number of participants within each subgroup limits the strength of any conclusions that can be drawn. The variability observed across cohorts is likely influenced by differences in clinical characteristics, disease severity, inflammatory phenotypes, and treatment exposure. Moreover, ER stress and UPR signalling are highly dynamic processes that respond differently depending on the severity and chronicity of cellular stress. Mild ER stress activates adaptive UPR pathways that restore ER homeostasis and resolve protein misfolding. In contrast, chronic or severe ER stress, such as that seen in steroid‐resistant severe asthma, favours pathways associated with ER‐associated protein degradation (ERAD), suppression of protein translation, and, if unresolved, the initiation of apoptotic signalling. These context‐dependent regulatory mechanisms contribute to the complexity of interpreting ER stress/UPR signatures across heterogeneous patient groups. Given these biological and clinical differences, the variability in findings between cohorts cannot be fully explained at this stage. Further studies, ideally incorporating additional independent cohorts and complementary sample types, will be necessary to provide clearer clarification of these relationships.

We also observed that several ERS/UPR genes correlate with some of the six‐gene signature genes described by Baines et al. (JACI, 2014) [66]. The CPA3 gene, which is expressed at relatively low levels in steroid non‐responders, showed a strong negative correlation with the ATF6 and SEL1L genes. The genes ALPL and CXCR2, which are predominantly expressed in the steroid‐insensitive neutrophilic asthma subgroup, positively correlated with ATF6, XBP1, and DNAJB9 genes. These correlation patterns further support that the ERS/UPR gene signature is associated with steroid insensitivity in asthma.

Although our sputum correlation findings are promising, it is important to acknowledge that the sputum microarray dataset used in this study predominantly reflects gene expression changes arising from airway immune cell populations rather than epithelial cells. Sputum‐derived transcriptomic profiles are typically enriched for neutrophils, macrophages, and other inflammatory cells, with epithelial cells often under‐represented. Because ICS exert potent immunomodulatory effects on these immune cell types, the observed associations between ER stress markers and glucocorticoid receptor‐related pathways in the sputum dataset likely represent immune cell‐driven mechanisms rather than direct epithelial contributions. Therefore, while our in vitro findings using airway epithelial models provide mechanistic insight into epithelial ER stress and corticosteroid responsiveness, interpretations linking these findings to the sputum data should be made cautiously.

Our findings provide compelling evidence that facilitating ER‐protein folding capacity by treating with 4‐PBA restored ERS‐induced corticosteroid insensitivity. We further confirmed these findings by testing in well‐established mouse models of SRAAD. We have previously shown that our model of SRAAD also exhibits upregulated ERS and UPR markers [26]. Taken together, our pre‐clinical findings demonstrate that facilitating ER protein folding using chemical chaperones could be an effective add‐on treatment to inhaled corticosteroids that may enhance corticosteroid sensitivity and efficacy in the airways of patients with severe asthma. Despite promising results, our pre‐clinical models provide limited evidence to draw definitive conclusions on the molecular basis of ER stress/UPR‐ and corticosteroid responsiveness in vivo. Our gene expression assays showed minimal transcriptional changes across treatment groups and were difficult to interpret (Figures S5 and S6). This likely reflects the use of bulk lung tissue, in which cell‐type–specific responses are diluted, as well as the fact that the lungs were lavaged prior to tissue collection, further reducing the suitability of these samples for detecting low‐abundance transcripts. RNA obtained from BAL cells was also insufficient in quality and yield to support reliable analysis. Although our in vivo model assesses the functional physiological impact of 4‐PBA, additional targeted molecular profiling would require a dedicated animal study. Future work using appropriately collected tissue or cell‐type‐specific approaches (e.g., sorted populations or single‐cell methods) will be necessary to more precisely resolve ERS/UPR and corticosteroid‐related pathways in this context.

Although our findings in this study are encouraging, the link between corticosteroid insensitivity and ERS or protein misfolding is unclear. Consistent with our findings, another study conducted with cell lines from acute lymphoblastic leukaemia showed increased corticosteroid resistance with ERS induction; however, the mechanism was not assessed [67]. When the ER is overwhelmed by nascent proteins and when the protein folding demand is increased, ERS activates the UPR, which increases the transcription of chaperones and actively recruits them into the ER to enhance protein folding capacity. This potentially may affect the availability of accessory chaperone proteins associated with GR‐HSP90 heterocomplexes that reside in the cytoplasm. Increased demand in the ER may cause these proteins to migrate into the ER for folding, which could result in disruption of GR‐HSP90 heterocomplex formation. Improper GR‐HSP90 heterocomplex formation reduces binding affinity to corticosteroids, leading to corticosteroid resistance. Enhancing ER protein folding capacity with an external chemical chaperone (4‐PBA) alleviates ERS and may allow accessory chaperone proteins in GR‐HSP90 heterocomplexes to reside in the cytoplasm, allowing proper GR‐corticosteroid binding. As our findings from sputum cell correlation analysis suggest, it is also possible that the increased ERS simply downregulate the co‐chaperone expression in the GR‐HSP90 heterocomplex and thus is unavailable for GR folding and transcriptionally inactive for corticosteroid binding. Despite these suggestions providing plausible mechanistic explanations, further detailed studies are required to elucidate the precise mechanisms involved.

Taken together, our findings strongly suggest that ERS and protein misfolding play a direct role in corticosteroid insensitivity in the airways of patients with severe asthma. Enhancing ER protein folding capacity using FDA‐approved 4‐PBA could be a novel therapeutic strategy to treat severe corticosteroid‐insensitive asthma.

## Author Contributions

Conceptualisation: Prabuddha S. Pathinayake, Alexandra C. Brown, Philip M. Hansbro, Katherine J. Baines, Richard Y. Kim, Jay C. Horvat and Peter A.B. Wark. Data curation: Prabuddha S. Pathinayake, Alexandra C. Brown, Nikhil T. Awatade, Katherine J. Baines, Richard Y. Kim, Jay C. Horvat and Peter A.B. Wark. Formal analysis: Prabuddha S. Pathinayake, Alexandra C. Brown, Nikhil T. Awatade, Katherine J. Baines, Richard Y. Kim, Nathan W. Bartlett, Jay C. Horvat and Peter A.B. Wark. Investigation: Prabuddha S. Pathinayake, Alexandra C. Brown, Nikhil T. Awatade, Philip M. Hansbro, Katherine J. Baines, Richard Y. Kim, Jay C. Horvat and Peter A.B. Wark. Methodology: Prabuddha S. Pathinayake, Alexandra C. Brown, Nikhil T. Awatade, Katherine J. Baines, Richard Y. Kim. Project administration: Prabuddha S. Pathinayake, Philip M. Hansbro, Katherine J. Baines, Richard Y. Kim, Nathan W. Bartlett, Jay C. Horvat and Peter A.B. Wark. Resources: Philip M. Hansbro, Katherine J. Baines, Richard Y. Kim, Nathan W. Bartlett, Jay C. Horvat and Peter A.B. Wark. Supervision: Philip M. Hansbro, Katherine J. Baines, Richard Y. Kim, Nathan W. Bartlett, Jay C. Horvat and Peter A.B. Wark. Software: Prabuddha S. Pathinayake, Alexandra C. Brown and Nikhil T. Awatade. Validation: Prabuddha S. Pathinayake, Alexandra C. Brown, Nikhil T. Awatade, Katherine J. Baines and Jay C. Horvat. Visualisation: Prabuddha S. Pathinayake, Alexandra C. Brown and Nikhil T. Awatade. Writing – original draft: Prabuddha S. Pathinayake, Alexandra C. Brown, Nikhil T. Awatade, Philip M. Hansbro, Katherine J. Baines, Richard Y. Kim, Jay C. Horvat and Peter A.B. Wark. Writing – review and editing: Prabuddha S. Pathinayake, Alexandra C. Brown, Katherine J. Baines, Richard Y. Kim, Nathan W. Bartlett, Jay C. Horvat and Peter A.B. Wark.

## Funding

This research was funded by the National Health and Medical Research Centre (NHMRC), Australia (APP2002953).

## Conflicts of Interest

The authors declare no conflicts of interest.

## Supporting information


**Figure S1:** Annexin V apoptosis detection assay (Thermo Fisher Scientific) was used to detect the live, early‐apoptosis, late‐apoptosis and necrotic cell populations. BCi‐NS1.1 cells were treated with tunicamycin, thapsigargin for 1 h and cultured for 2, 12 or 48 h. Cells treated with 0.1 mM H_2_O_2_ for 12 h were used as the positive control. Cells cultured with BEGM culture media were used as a negative control (*n* = 4).
**Figure S2:** Correlation matrix analysis of sputum microarray data shows the Spearman correlation coefficient between (A). ERS/UPR and co‐chaperone of GR‐HSP90 complex (B). ERS/UPR and ICS responsive genes (C). ERS/UPR and Asthma signature genes. *p* values were obtained from pair‐wise analysis, and significant correlations are indicated with asterisks.
**Figure S3A:** Correlation matrix analysis of U‐BIOPRED severe asthma, non‐smoker, Sputum cohort shows the Spearman correlation coefficient between ERS/UPR, chaperone components of GR‐HSP90 complex, GR‐signalling, TH2, and TH1/17 genes. ERS/UPR vs. chaperone components of GR‐HSP90 complex genes have been demarcated with a yellow colour square for direct comparison with the sputum microarray data.
**Figure S3B:** Correlation matrix analysis of U‐BIOPRED mild–moderate asthma, non‐smoker, Sputum cohort shows the Spearman correlation coefficient between ERS/UPR, chaperone components of GR‐HSP90 complex, GR‐signalling, TH2, and TH1/17 genes. ERS/UPR vs. chaperone components of GR‐HSP90 complex genes have been demarcated with a yellow colour square for direct comparison with the sputum microarray data.
**Figure S3C:** Correlation matrix analysis of U‐BIOPRED severe asthma, non‐smoker, Sputum, eosinophilic phenotype (TH2) cohort shows the Spearman correlation coefficient between ERS/UPR, chaperone components of GR‐HSP90 complex, GR‐signalling, TH2, and TH1/17 genes. ERS/UPR vs. chaperone components of GR‐HSP90 complex genes have been demarcated with a yellow colour square for direct comparison with the sputum microarray data.
**Figure S3D:** Correlation matrix analysis of U‐BIOPRED severe asthma, non‐smoker, Sputum, neutrophilic phenotype (TH1) cohort shows the Spearman correlation coefficient between ERS/UPR, chaperone components of GR‐HSP90 complex, GR‐signalling, TH2, and TH1/17 genes. ERS/UPR vs. chaperone components of GR‐HSP90 complex genes have been demarcated with a yellow colour square for direct comparison with the sputum microarray data.
**Figure S4:** Primary bronchial epithelial cells (*n* = 4) differentiated at the air–liquid interface (ALI) were challenged with TH1/17 cytokine mix containing 10 ng/mL IL‐17A, 5 ng/mL IFN‐ɣ and 1 ng/mL TNF for 10 days to induce corticosteroid insensitivity. The basal media containing the cytokine mix was replenished every 2 days. At the end of the cytokine treatment, ALIs were treated with dexamethasone (Dex, 100 nM) alone or with 4‐PBA (2 mM) for 12 h. ERS/UPR genes (A). DDIT3 (B). EIF2AK3 (C). EDEM1 (D). BIP and (E) HERPUD1 were measured by qPCR.
**Figure S5:** ERS/UPR gene expression of whole lung tissues of *Chlamydia muridarum* (Cmu)‐induced severe corticosteroid‐resistant allergic airways disease (SRAAD). Mice were sensitised with intraperitoneal (i.p.) injection of ovalbumin and aluminium hydroxide (Ova, Day 0) and subsequently challenged intranasally (i.n.) with Ova to establish AAD (Days 12, 13, 33 and 34). SRAAD was induced by infecting mice i.n. with Cmu (Day 14). The effects of i.n. treatments with dexamethasone (Dex; 2 mg/kg) or 4‐PBA (8 mg/kg) alone or in combination were investigated (Days 32–34). Controls were sham‐sensitised with i.p injection of saline and aluminium hydroxide (Sal, Day 0), sham‐infected with sucrose phosphate glutamate buffer (Day 14) and/or sham‐treated with PBS (Days 32–34). (B). At Day 35, mice were euthanised, and whole lung tissues were collected. Gene expression of ERS/UPR genes (A). XBP1 (B) CHOP (C). BiP (D). EDEM1 (E) ATF4 and (F) CREB3L1 was measured by qPCR.
**Figure S6:** ERS/UPR gene expression of whole lung tissues of non‐typeable *Haemophilus influenzae* (Hinf)‐induced severe corticosteroid‐resistant allergic airways disease (SRAAD). Mice were sensitised with intraperitoneal (i.p.) injection of ovalbumin and aluminium hydroxide (Ova, Day 0) and subsequently challenged intranasally (i.n.) with Ova to establish AAD (Days 12, 13, 33 and 34). SRAAD was induced by infecting mice i.n. with Hinf (Day 14). The effects of i.n. treatments with dexamethasone (Dex; 2 mg/kg) or 4‐PBA (8 mg/kg) alone or in combination were investigated (Days 32–34). Controls were sham‐sensitised with i.p injection of saline and aluminium hydroxide (Sal, Day 0), sham‐infected with PBS (Day 14) and/or sham‐treated with PBS (Days 32–34). At Day 35, mice were euthanised, and whole lung tissues were collected. Gene expression of ERS/UPR genes (A) XBP1 (B) CHOP (C) BiP (D). EDEM1 (E) ATF4 and (F). CREB3L1 was measured by qPCR.
**Figure S7:** Schematic diagrams show the treatment timeline for each treatment for the *Chlamydia muridarum* (Cmu)‐induced severe corticosteroid‐resistant allergic airways disease (SRAAD) model.
**Figure S8:** Schematic diagrams show the treatment timeline for each treatment for the non‐typeable *Haemophilus influenzae* (Hinf)‐induced severe corticosteroid‐resistant allergic airways disease (SRAAD)model.

## Data Availability

The data that support the findings of this study are openly available in Figshare at https://figshare.com/account/items/29036624/edit, reference number https://doi.org/10.6084/m9.figshare.29036624.
