# Supplementary material for: Resolving Endoplasmic Reticulum‐Protein Misfolding Restores Corticosteroid Sensitivity in Experimental Models of Severe Asthma
Source: Allergy. 2026 Apr 26;81(9):3221–35. doi: 10.1111/all.70353 (PMC13569705; doi:10.1111/all.70353)
Supplement: Supplementary file 1 [file ALL-81-3221-s001.docx]

**Resolving endoplasmic reticulum-protein misfolding restores corticosteroid sensitivity in experimental models of severe asthma.**

Prabuddha S. Pathinayake^1^, Alexandra C. Brown^2^, Nikhil T. Awatade^3^, Philip M. Hansbro^5^, Katherine J. Baines^2^, Richard Y. Kim^2,8^, Nathan W. Bartlett^1^, Jay C. Horvat^2^ and Peter A.B. Wark*^2, 4, 6, 7^

Affiliations:

^1^Infection Research Program, Hunter Medical Research Institute and School of Biomedical Sciences and Pharmacy, University of Newcastle, Newcastle, New South Wales, Australia.

^2^Immune Health Program, Hunter Medical Research Institute and School of Biomedical Sciences and Pharmacy, University of Newcastle, Newcastle, New South Wales, Australia.

^3^Immune Health Program, Hunter Medical Research Institute and School of Medicine and Public Health, University of Newcastle, Newcastle, New South Wales, Australia.

^4^Department of Respiratory and Sleep Medicine, John Hunter Hospital, Newcastle, New South Wales, Australia.

^5^Centre for Inflammation, Centenary Institute, and University of Technology Sydney, Faculty of Science, School of Life Sciences, Sydney, New South Wales, Australia.

^6^ School of Translational Medicine, Monash University, Melbourne, Victoria, Australia.

^7^AIRMED Alfred Health, Melbourne, Victoria, Australia.

^8^University of Technology Sydney, Faculty of Science, School of Life Sciences, Sydney, New South Wales, Australia.

*Correspondence:

Address: AIRMED Alfred Health, Melbourne, Victoria, Australia

e-mail: *peter.wark@monash.edu*

Tel: 03 90763698

Short title: Restoring steroid sensitivity in severe asthma

Funding statement: This research was funded by the National Health and Medical Research Centre (NHMRC), Australia (APP2002953).

Declaration of interests: No conflict of interest.

**Acknowledgements**

PMH is funded by a Fellowship and grants from the National Health and Medical Research Council (NHMRC) of Australia (1175134, 2010287, 2008937, 2015613), Australian Research Council (200101058, 230101156) and by UTS

**Supplementary Figures:**

**Supplementary Figure 1**. Annexin V apoptosis detection assay (Thermo Fisher Scientific) was used to detect the live, early-apoptosis, late-apoptosis and necrotic cell populations. BCi-NS 1.1 cells were treated with tunicamycin, thapsigargin for 1hr and cultured for 2, 12 or 48 hours. Cells treated with 0.1mM H2O2 for 12 hours were used as the positive control. Cells cultured with BEGM culture media were used as a negative control. (n=4)

**Supplementary Figure 2.** Correlation matrix analysis of sputum microarray data shows the Spearman correlation coefficient between (A). ERS/UPR and co-chaperone of GR-HSP90 complex (B). ERS/UPR and ICS responsive genes (C). ERS/UPR and Asthma signature genes. P values were obtained from pair-wise analysis, and significant correlations are indicated with asterisks.

**Supplementary Figure 3. (A)** Correlation matrix analysis of U-BIOPRED severe asthma, non-smoker, Sputum cohort shows the Spearman correlation coefficient between ERS/UPR, chaperone components of GR-HSP90 complex, GR-signalling, TH2, and TH1/17 genes. ERS/UPR vs chaperone components of GR-HSP90 complex genes have been demarcated with a yellow colour square for direct comparison with the sputum microarray data.

B

**Supplementary Figure 3. (B)** Correlation matrix analysis of U-BIOPRED mild-moderate asthma, non-smoker, Sputum cohort shows the Spearman correlation coefficient between ERS/UPR, chaperone components of GR-HSP90 complex, GR-signalling, TH2, and TH1/17 genes. ERS/UPR vs chaperone components of GR-HSP90 complex genes have been demarcated with a yellow colour square for direct comparison with the sputum microarray data.

C

**Supplementary Figure 3. (C)** Correlation matrix analysis of U-BIOPRED severe asthma, non-smoker, Sputum, eosinophilic phenotype (TH2) cohort shows the Spearman correlation coefficient between ERS/UPR, chaperone components of GR-HSP90 complex, GR-signalling, TH2, and TH1/17 genes. ERS/UPR vs chaperone components of GR-HSP90 complex genes have been demarcated with a yellow colour square for direct comparison with the sputum microarray data.

D

**Supplementary Figure 3. (D)** Correlation matrix analysis of U-BIOPRED severe asthma, non-smoker, Sputum, neutrophilic phenotype (TH1) cohort shows the Spearman correlation coefficient between ERS/UPR, chaperone components of GR-HSP90 complex, GR-signalling, TH2, and TH1/17 genes. ERS/UPR vs chaperone components of GR-HSP90 complex genes have been demarcated with a yellow colour square for direct comparison with the sputum microarray data.

**Supplementary Figure 4.** Primary bronchial epithelial cells (n=4) differentiated at the air-liquid interface (ALI) were challenged with TH1/17 cytokine mix containing 10ng/mL IL-17A, 5ng/mL IFN-ɣ and 1ng/mL TNF for 10 days to induce corticosteroid insensitivity. The basal media containing the cytokine mix was replenished every two days. At the end of the cytokine treatment, ALIs were treated with dexamethasone (Dex, 100nM) alone or with 4-PBA (2mM) for 12 hrs. ERS/UPR genes (A). DDIT3 (B). EIF2AK3 (C). EDEM1 (D). BIP and (E) HERPUD1 were measured by qPCR

**Supplementary Figure 5.** ERS/UPR gene expression of whole lung tissues of Chlamydia muridarum (Cmu)-induced severe corticosteroid-resistant allergic airways disease (SRAAD). Mice were sensitised with intraperitoneal (i.p.) injection of ovalbumin and aluminium hydroxide (Ova, day 0) and subsequently challenged intranasally (i.n.) with Ova to establish AAD (days 12, 13, 33 and 34). SRAAD was induced by infecting mice i.n. with Cmu (day 14). The effects of i.n. treatments with dexamethasone (Dex; 2mg/kg) or 4-PBA (8mg/kg) alone or in combination were investigated (days 32-34). Controls were sham-sensitized with i.p injection of saline and aluminium hydroxide (Sal, day 0), sham-infected with sucrose phosphate glutamate buffer (day 14) and/or sham-treated with PBS (days 32-34). (B). At day 35, mice were euthanised, and whole lung tissues were collected. Gene expression of ERS/UPR genes (A). XBP1 (B).CHOP (C). BiP (D). EDEM1 (E).ATF4 and (F). CREB3L1 was measured by qPCR.

**Supplementary Figure 6.** ERS/UPR gene expression of whole lung tissues of non-typeable Haemophilus influenzae (Hinf)-induced severe corticosteroid-resistant allergic airways disease (SRAAD). Mice were sensitised with intraperitoneal (i.p.) injection of ovalbumin and aluminium hydroxide (Ova, day 0) and subsequently challenged intranasally (i.n.) with Ova to establish AAD (days 12, 13, 33 and 34). SRAAD was induced by infecting mice i.n. with Hinf (day 14). The effects of i.n. treatments with dexamethasone (Dex; 2mg/kg) or 4-PBA (8mg/kg) alone or in combination were investigated (days 32-34). Controls were sham-sensitized with i.p injection of saline and aluminium hydroxide (Sal, day 0), sham-infected with PBS (day 14) and/or sham-treated with PBS (days 32-34). At day 35, mice were euthanised, and whole lung tissues were collected. Gene expression of ERS/UPR genes (A). XBP1 (B).CHOP (C). BiP (D). EDEM1 (E).ATF4 and (F). CREB3L1 was measured by qPCR.

**Supplementary Figure 7.** Schematic diagrams show the treatment timeline for each treatment for the Chlamydia muridarum (Cmu)-induced severe corticosteroid-resistant allergic airways disease (SRAAD) model.

**Supplementary Figure 8.** Schematic diagrams show the treatment timeline for each treatment for the non-typeable Haemophilus influenzae (Hinf)-induced severe corticosteroid-resistant allergic airways disease (SRAAD)model.

Adjusted P values for the Spearman correlation matrix analysis for the U-BIOPRED severe asthma non-smoker cohort (n=49). Correlations between ERS/UPR and GR-HSP90 complex, ERS/UPR and Glucocorticoid signalling are demarcated in a purple colour square. P values/R values significant are highlighted in yellow.
